# Kinematic Parameters Following Pilon Fracture Treatment with the Ilizarov Method

**DOI:** 10.3390/jcm11102763

**Published:** 2022-05-13

**Authors:** Paweł Wietecki, Łukasz Pawik, Felicja Fink-Lwow, Artur Leśkow, Radosław Górski, Malwina Pawik, Jarosław Olech, Krzysztof Klepacki, Patryk Kuliński, Paweł Reichert, Piotr Morasiewicz

**Affiliations:** 1 Department of Trauma and Hand Surgery, Wroclaw Medical University, 50-367 Wrocław, Poland; dooktur@wp.pl (P.W.); pawel.reichert@umed.wroc.pl (P.R.); 2 Department of Physiotherapy in Motor Disorders and Dysfunctions, University School of Physical Education in Wroclaw, 51-612 Wroclaw, Poland; lukaszpawik@gmail.com; 3 Department of Massage and Physical Therapy, Faculty of Physiotherapy, Wroclaw University of Health and Sport Sciences, 51-612 Wrocław, Poland; felicitas1@wp.pl (F.F.-L.); malwina.pawik@gmail.com (M.P.); 4 Department of Orthopedics and Musculoskeletal Traumatology, Medical University of Warsaw, 02-005 Warsaw, Poland; artur.leskow@gmail.com (A.L.); radoslaw.gorski@wp.pl (R.G.); 5 Orthopedic Surgery Department, Provincial Specialist Hospital in Legnica, 59-220 Legnica, Poland; o.jaroslaw@yahoo.com (J.O.); krzysztof.klepacki90@gmail.com (K.K.); 6 Lower Silesia Specialist Hospital, 54-049 Wrocław, Poland; kulinski.patryk@gmail.com; 7 Institute of Medical Sciences, Department of Orthopaedic and Trauma Surgery, University Hospital in Opole, University of Opole, 45-401 Opole, Poland

**Keywords:** kinematic, range-of-motion, pilon fracture, Ilizarov method

## Abstract

Background: The purpose of our study was to analyze kinematic parameters following pilon fracture treatment with the Ilizarov method. Methods: Our study assessed kinematic parameters of gait in 23 patients with pilon fractures treated with the Ilizarov method. Patients had completed their treatment 24–48 months prior to measurements. The range-of-motion values in the non-operated limb (NOL) and operated limb (OL) were compared. Kinematic parameters were measured using the Noraxon MyoMOTION System. Results: We observed no significant differences in hip flexion, hip abduction, or knee flection between the OLs and NOLs in patients after treatment with the Ilizarov method. We observed significant differences in the ranges of ankle dorsiflexion, inversion, and abduction (*p* < 0.001; *p* < 0.001; *p* < 0.003, respectively) between the OLs and the NOLs. Conclusion: Following pilon fracture treatment with the Ilizarov method, we observed no differences in terms of knee or hip joint mobility between the OL and the NOL, whereas the range of motion in the ankle joint of the OL was significantly limited. The treatment of pilon fractures with the Ilizarov method does not ensure the complete normalization of ankle joint kinematic parameters. Therefore, intense personalized rehabilitation of the ankle joint is recommended.

## 1. Introduction

One of the established treatment methods for the type of distal tibia fractures called pilon fractures is Ilizarov fixation. This method is chosen particularly for extensive injuries involving soft tissue damage and compound fractures that result from considerable forces [1,2,3,4,5,6,7,8,9,10,11,12].

Such injuries may be additionally complicated by infections and delayed soft-tissue healing, which may hinder treatment and rehabilitation [1,3,5,7,10]. Notably, the management of pilon fractures requires dealing with soft tissue injuries besides merely performing fracture reduction and bone fragment fixation. The risk of complications precipitated the development of closed techniques (the Ilizarov method) and external fixators as an alternative to internal fixation techniques [5,7,10]. According to some authors, the Ilizarov method yields functional outcomes comparable to those achieved with internal fixation, with significantly lower infection rates. Nonetheless, the reported outcomes vary, with some orthopedic surgeons having observed good treatment outcomes in pilon fractures with the use of external fixators as the final treatment technique [1,2,3,6,8,10,11] and some studies failing to demonstrate beneficial treatment outcomes with the use of external fixation for this type of injury [1,4,5,10,12].

From the patients’ point of view, the key goal of the treatment is to improve everyday functioning, which determines the quality of life. Therefore, any analysis of the final treatment outcomes in musculoskeletal pathologies should be multifaceted and include not only clinical and radiographic examination but also an assessment of lower limb biomechanical parameters, with a particular emphasis on detecting any deficits in the injured limb, in comparison with the healthy limb [13,14,15,16,17,18,19,20]. Physiological kinematic parameters of gait, including the ranges of joint movement, should be symmetrical in both limbs [13,14,16,17]. Patients treated for distal tibial fractures (pilon fractures) may experience limitations in the range of ankle movement, malunion, ankle joint instability, and the resulting chronic pain and posttraumatic degenerative changes in the ankle joint and pain in other lower limb joints [1,3,5,7,10,11,12]. Such complications may be due to the complexity of the original injury, extensive soft-tissue injuries, the selected treatment technique, or the quality of the final therapeutic procedure. Assessing the range of motion (particularly at the ankle joint) following pilon fracture treatment is important, since it helps evaluate clinical and functional outcomes and determine which ranges of motion have become limited [13,14,15,16,17,18,19,20].

The assessment of biomechanical gait parameters in patients after pilon fracture treatment has been explored to a limited extent in the available literature. Some studies have assessed the biomechanical force (load) distribution throughout the individual areas of the foot with the use of pedobaric platforms, while other studies have focused on gait speed, step length, and cadence [11,12,14]. To our knowledge, the ranges of hip, knee, or ankle joint motion during gait following treatment of pilon fractures have never been assessed. Some authors used a goniometer to measure the range of ankle motion at rest in patients after pilon fracture [21,22,23,24]. In our study, we used the Noraxon MyoMOTION System, which provides very accurate, repeatable, and objective readings on joint motion in real time [25,26,27,28].

The purpose of our study was to analyze kinematic parameters following pilon fracture treatment with the Ilizarov method, and to determine if the Ilizarov method restores normal joint function and improves knee and hip mobility, thus improving gait symmetry between the operated and healthy limb.

## 2. Methods

Our study assessed the kinematic parameters of gait in 23 patients with pilon fractures treated with the Ilizarov method. After treatment, none of the evaluated patients required limb lengthening or axis correction. None of the patients developed a permanent limb deformity. After treatment, both lower limbs of all treated patients were of equal length or there was a shortening of less than 1 cm. The study inclusion criteria were distal tibial fracture (pilon fracture) treatment with an Ilizarov fixator, clinical and radiographic evidence of complete bone union, no musculoskeletal injuries in the contralateral limb, complete clinical and radiographic records as well as gait assessment records, a follow-up period of at least 2 years after treatment completion, and written informed consent. The exclusion criteria were pilon fractures treated with other techniques, a follow-up period of less than 2 years or more than 4 years after treatment completion, lower limb comorbidities, incomplete clinical or radiographic records, and a lack of gait assessment with the use of the Noraxon MyoMOTION System.

Our study was approved by the University Senate Research Bioethics Committee, University School of Physical Education, Wroclaw (case No. 5/2020) and was conducted in accordance with the Code of Ethics of the World Medical Association (Declaration of Helsinki) for experiments involving humans. The study was conducted between 2019 and 2020. Following the application of the inclusion and exclusion criteria, a total of 23 patients (seven females and sixteen males; age 54.9 ± 16.4 years; height 170.0 ± 11 cm; body weight 81.4 ± 14.0 kg; body mass index (BMI) 28.1 ± 3.9 kg/m^2^) were included in the study. The patients had completed their treatment 24–48 months prior to MyoMOTION measurements and had completed their entire physiotherapy protocol, including a comprehensive, personalized rehabilitation regimen (i.e., one consisting of individual rehabilitation, scar and muscle fascia therapy, as well as balance exercises and active lower limb exercises).

All patients evaluated in our study had undergone closed reduction and Ilizarov fixation. The Ilizarov external fixators were composed of 3 or 4 rings secured to the fibula and tibia via Kirschner wires. Ambulation with 2 elbow crutches with partial weight-bearing was initiated on postoperative day 1. The patients gradually increased weight bearing until full weight bearing on the operated limb (OL) 2–3 months after treatment initiation. The Ilizarov fixator was removed after an orthopedic clinical examination (in the absence of pain or pathological mobility at the fracture site) and a radiographic assessment (the presence of at least 3 out of 4 cortices) of bone union. Once the Ilizarov fixator was removed, the patients walked with the help of 2 elbow crutches and partial weight-bearing over a period of 4 weeks and underwent a pre-planned rehabilitation regimen.

The rehabilitation protocol was adjusted for the individual patients’ condition and their current functional capacity. The regimen included active hip, knee, and ankle exercises within pain tolerance and isometric exercises (particularly those involving the vastus medialis oblique (VMO) muscle and the gluteus maximus and medius muscles) for a period of 4 weeks after surgery. The regimen also included fascia therapy, proprioception exercises, and scar mobilization, which started 2 weeks after the decision to dismantle the external fixator. At the same time, efforts were made to help the patient walk with two elbow crutches, both over level surfaces and stairs. During the subsequent 4–6 weeks, the rehabilitation exercises progressed to the new stage and were complemented by strengthening exercises of the non-operated limb (NOL) in a sitting position, balance exercises, strengthening exercises with the use of resistance bands, and manual therapy. Subsequent stages of rehabilitation (8–10 weeks after surgery) involved the use of strengthening exercises in a standing position, balance exercises, and the progression of exercises from the earlier stages, in order to achieve optimal range of motion and muscle strength.

The range-of-motion values in the NOL and operated limb (OL) were compared. Kinematic parameters were measured using the Noraxon MyoMOTION System (Scottsdale, AZ, USA) composed of a set of 1–16 inertial sensors.

**Figure 1:**
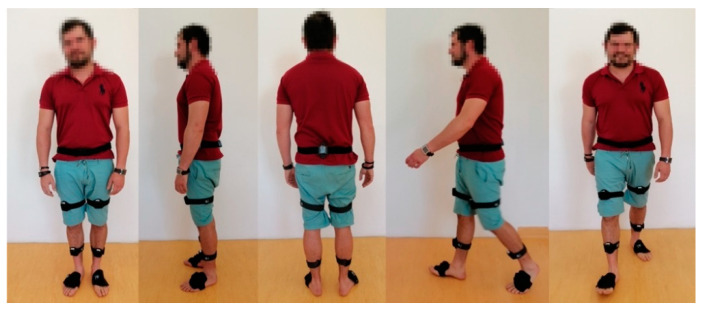
Patient with sensors during examination.

Following the manufacturer’s instructions, Noraxon MyoMOTION System inertial sensors were placed on the sacrum, on the thigh (anterior part of the quadriceps muscle, 3–5 cm above the kneecap), on the leg (on the front side, halfway between the ankles and the kneecap), and on the foot (on the dorsal side, slightly below the ankles) (Figure 1). All sensors were attached by the same technician with elastic adhesive tape and special strips (each strap had a pocket for an inertia sensor). Calibration was performed in a vertical position in order to determine the value of the 0° angle in the joints. The angle values were recorded with an accuracy of 0.1° and analyzed statistically.

We measured hip flexion and abduction, knee flexion, and ankle dorsiflexion, inversion, and abduction during a ten-meter walk along a straight line. 

Each person performed at least four repetitions, and the mean values from at least three complete, correct walks were used in the statistical analysis. In order to prepare the patient for the study, the first test was treated as a mock test and was not included in the statistical analysis.

### Statistical Analysis

Continuous variables were first analyzed for a normal distribution using the Kolmogorov–Smirnov test with Lilliefors correction. The data exhibiting a normal distribution were presented as means ± standard deviations (SDs), and an unpaired Student’s *t*-test was used to test the differences between the OLs and NOLs. In the case of data that did not pass the normality test, the significance of differences was analyzed using the Mann–Whitney *U*-test, and the data were expressed as the medians and 5th to 95th percentile ranges. The level of statistical significance was set at *p* < 0.05. All analyses were conducted using the SigmaPlot v.13 statistics package (Systat Software, San Jose, CA, USA).

## 3. Results

**Figure 2:**
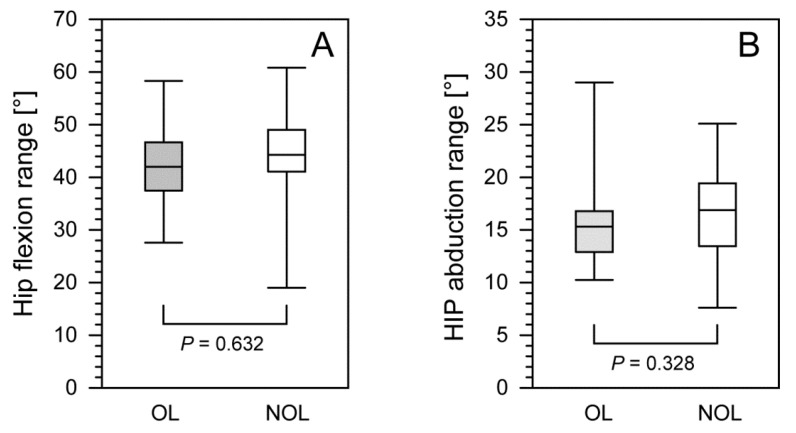
The comparison of hip flexion range (**panel A**) and hip abduction range (**panel B**) between the operated and non-operated limbs in patients after treatment with the Ilizarov method. The lower border of each box indicates the 25th percentile, the line within the box marks the median, and the upper border of each box indicates the 75th percentile. The whiskers above and below the boxes indicate the 90th and 10th percentile, respectively. Filled box, operated limb (OL); white box, non-operated limb (NOL).

**Figure 3:**
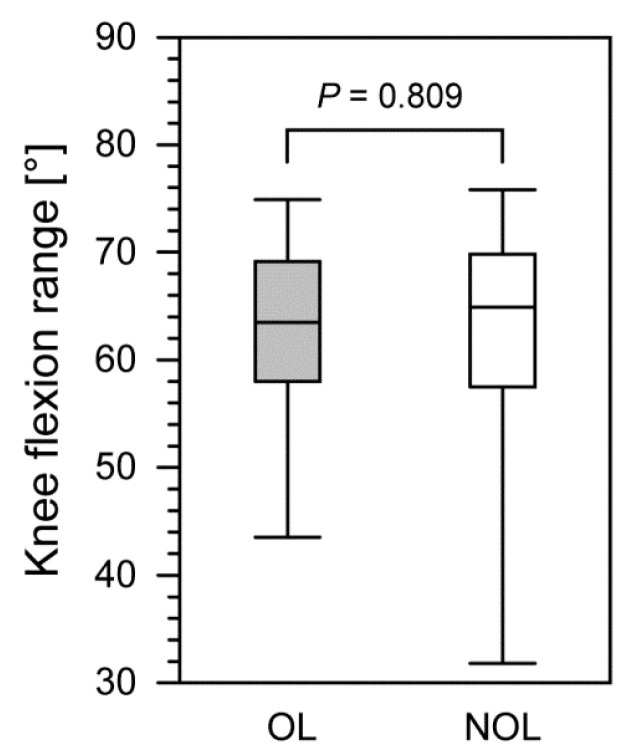
The comparison of knee flexion range between the operated and non-operated limbs in patients after treatment with the Ilizarov method. The lower border of each box indicates the 25th percentile, the line within the box marks the median, and the upper border of each box indicates the 75th percentile. The whiskers above and below the boxes indicate the 90th and 10th percentile, respectively. Filled box, operated limb (OL); white box, non-operated limb (NOL).

We observed no significant differences in hip flexion (i.e., minimum, maximum, or range), hip abduction (i.e., minimum, maximum, or range) (Table 1), or knee flexion (i.e., minimum, maximum, or range) (Table 2) between the OLs and NOLs in patients after treatment with the Ilizarov method. Furthermore, 24–48 months after Ilizarov treatment, we compared the OLs and NOLs in terms of the ranges of hip flexion (*p* = 0.632), hip abduction (*p* = 0.328) (Figure 2), and the knee flexion (*p* = 0.809) (Figure 3).

**Figure 4:**
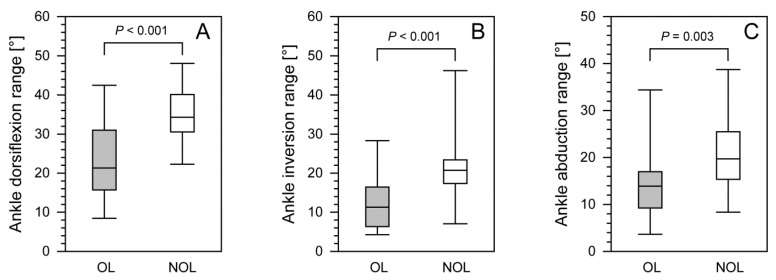
The comparison of ankle dorsiflexion range (**panel A**), ankle inversion range (**panel B**), and ankle abduction range (**panel C**) between operated and non-operated limbs for patients after treatment with the Ilizarov method. The lower border of each box indicates the 25th percentile, the line within the box marks the median, and the upper border of each box indicates the 75th percentile. The whiskers above and below the boxes indicate the 90th and 10th percentile, respectively. Filled box, operated limb (OL); white box, non-operated limb (NOL).

We observed significant differences in the ranges of ankle dorsiflexion, inversion, and abduction (*p* < 0.001; *p* < 0.001; *p* < 0.003, respectively) between the operated limbs (OLs) and the non-operated limbs (NOLs) in patients after treatment with the Ilizarov method (Figure 4).

We found significant differences in minimum and maximum ankle dorsiflexion (*p* = 0.011, *p* < 0.001), ankle inversion (*p* = 0.011; *p* = 0.005), and minimum ankle abduction *p* = 0.004 (Table 3).

## 4. Discussion

In our study, we thoroughly assessed the ranges of joint motion following pilon fracture treatment with the Ilizarov method and compared the mobility of the OLs and the NOLs. We observed a significantly decreased mobility of the OL in comparison with that of the NOL, in terms of all evaluated ankle joint movements (i.e., ankle dorsiflexion, inversion, and abduction). Therefore, the Ilizarov method in the treatment of pilon fractures failed to eliminate the deficit after at least 2 years following treatment completion. However, the ranges of hip and knee motion in the OL and NOL were comparable. In summary, by comparing the ranges of motion in the joints of the OL and NOL, we demonstrated that the Ilizarov method helps restore a normal range of motion at the knee and hip. Unfortunately, we did not observe an equally good outcome at the ankle joint. Other authors also demonstrated a limited range of motion at the ankle joint following pilon fracture treatment with various methods [21,23,24].

Nevertheless, our research, which was conducted in a homogeneous, though small, population, needs to be continued to demonstrate that the Ilizarov method can be used in the treatment of pilon fractures yielding kinematic parameter improvement, though not complete restoration. The Ilizarov method has been widely adopted by orthopedic surgeons for pilon fracture treatment due to inadequate treatment outcomes achieved with other methods, such as internal fixation with plates or intramedullary nails. The main objectives of pilon fracture treatment include achieving bone union, preserving limb length, restoring limb axis, and reestablishing the ability to walk. These objectives may be achieved by reconstructing the anatomical position of the bone fragments and normal joint congruency by using stable fixation, while preserving a good quality of adjacent soft tissues and making it possible to initiate limb mobility early [1,2,3,4,5,6,7,8,9,10,11,12]. These objectives proved too challenging to achieve with the use of internal fixation, hence the concept of closed treatment methods with the use of external fixators. The treatment methods employed so far have attempted to improve the patients’ quality of life (including their daily functioning in their normal environment and pain reduction) by normalizing gait parameters.

Normal gait and daily functioning require physiological joint mobility [12,14,18,19,27,28]. Abnormal kinematic parameters reflect both the treatment outcome and the quality of the employed treatment method [13,14,15,16,17,18,19,20]. Following treatment, the biomechanical parameters of the lower limbs are expected to improve along with the improvement in pain and joint range of motion [13,14,16,17] as well as limb symmetry. Like us, other authors have also demonstrated the usefulness of gait analysis in assessing Ilizarov method treatment outcomes [14,16,17,18,19,20].

To our best knowledge, there have been no studies evaluating the post-treatment range of hip, knee, and ankle motion during gait in patients with pilon fractures. These parameters are very important for orthopedic surgeons and rehabilitation specialists, since they identify the ranges of motion in the OL that require more attention during personalized rehabilitation and physical exercises to restore joint mobility following treatment and to eliminate any deficits with respect to the NOL. The Myomotion system helps measure the mobility of one joint or simultaneously measures mobility at all large joints during movement [25,26,27,28]. Unlike us, some authors who also assessed post-treatment gait in patients with pilon fractures did not analyze the range of joint motion [11,12,14]. Some other authors assessed ankle joint mobility at rest with low accuracy, with a hand-held goniometer [21,22,23,24].

Our analysis of joint mobility following pilon treatment with the Ilizarov method yielded results that were comparable with those of other authors. Vidyadhara and Rao analyzed 21 patients with pilon fractures treated with the Ilizarov method [21] and observed a limited range of ankle motion in the operated limb, reporting the range of ankle dorsiflexion of 5°–15° and plantar flexion of 5°–35° [21]. These results are consistent with ours. Osman et al. reported the range of ankle dorsiflexion of 0°–20° and plantar flexion of 5°–40° following pilon fractures treated with the Ilizarov method [22]. Firat et al. assessed 34 patients after pilon fracture treatment [23] and compared the treatment outcomes achieved with an external fixator and the Ilizarov method. The mean ankle dorsiflexion was 10.2° (4°–20°), and the mean plantar flexion was 25° (12°–45°) in the external fixator group, with the corresponding values in the Ilizarov group of 8.8° (3°–18°) and 12.4° (10°–50°), respectively [23]. Another similar study on the range of ankle joint motion following pilon fracture treatment was conducted by Ramos et al. [24]; those authors compared the treatment outcomes achieved in patients with extra-articular and intra-articular fractures and reported ankle dorsiflexion to be limited by >10° in three patients, and plantar flexion to be limited by >10° in seven patients [24]. In our study, the ranges of ankle dorsiflexion, inversion, and abduction were significantly lower in the OL than those in the NOL. The values of ankle joint motion in our patients were comparable with those from the cited literature [21,22,23,24].

In our patient population, the kinematic parameters of the hip and knee joints in the OL were just like the corresponding parameters in the NOL. However, the range of motion at the ankle joint was significantly worse in the OL than in the NOL. This may have resulted from several factors. One of those may have been the too-short rehabilitation treatment, which failed to improve the reduced ankle joint mobility. Another factor may have been the extensive scarring and adhesions in the skin, subcutaneous tissue, muscles, and tendons that developed in some patients, while the Achilles tendon is largely responsible for movement at the ankle joint [14,27]. Additionally, post-traumatic or degenerative joint deformities and pain may have contributed to the limited range of motion at the ankle joint [14,29,30]. Another factor that may have contributed to joint stiffness was the long period of immobilization with the external fixator [14,19]. Finally, the differences in ankle joint mobility may have also been due to compensatory mechanisms [17,25], which come from the fact that a change in the mobility of one joint causes compensatory changes in the mobility of other joints [17,25]. One factor that increases joint mobility is an increase in gait speed [27].

Physiological gait is generally known to be symmetrical [13,14,16,17]. However, slight differences in joint mobility between the left and right side of the body are normal [14,25,26]; for instance, the dominant limb may exhibit a greater range of joint motion [14,25,26]. Our study showed equal ranges of both hip and knee joint mobility in the OL and NOL. However, there was a loss of symmetry in joint mobility between the OL and the NOL in the ranges of ankle dorsiflexion, ankle inversion, and ankle abduction. All these differences in ankle joint mobility were statistically significant, which shows a lack of comparable ranges of motion at the ankle joint between the OL and the NOL 24–48 months after treatment.

Our study has some limitations. One of the weaknesses of the study was its retrospective nature, which is due to the impossibility of assessing kinetic parameters pre-operatively, since this was a population of patients with pilon fractures who were unable to walk or had considerable difficulty walking due to pain and pathological mobility at the ankle joint prior to receiving surgical treatment. It is worth noting that studies by other authors who assessed joint mobility are also retrospective in nature [19,21,22,23]. Another limitation of our study was the small sample size, which was a product of several factors: pilon fractures are a relatively rare injury, some patients with pilon fractures underwent other types of treatment (which disqualified them from study participation), and some patients lived too far from the center to attend gait assessment. However, most other studies assessing kinematic parameters included similarly small or even smaller patient populations [14,16,17,18,19,20,21,22,23,25,26,27,28]. In the future, we are planning a study comparing the kinematic parameters after the treatment of pilon fractures using the Ilizarov method and after open reposition and stabilization with a plate.

One of the strengths of our study was the homogeneous surgical protocol, homogeneous rehabilitation protocol, long follow-up period, and kinematic parameter assessment with an objective and highly accurate Myomotion system that yields repeatable results [25,26,27,28]. The effects of the Ilizarov treatment in terms of the kinematic parameters of the ankle joint were comparable with those reported by other authors.

In summary, we observed significant differences between the OL and the NOL in terms of ankle dorsiflexion, inversion, and abduction in patients with pilon fracture after 24–48 months of treatment completion using the Ilizarov method. There should be further studies in larger patient populations. In the future, we are planning to compare the kinematic parameters obtained from patients after pilon fracture treatment with Ilizarov fixation and those after treatment with internal fixation via plates. Additionally, our current observations suggest the need for intensive ankle joint rehabilitation following pilon fracture treatment.

## 5. Conclusions

1. Following pilon fracture treatment with the Ilizarov method, we observed no differences in terms of knee or hip joint mobility between the OL and the NOL, whereas the range of motion in the ankle joint of the OL was significantly limited.
2. The treatment of pilon fractures with the Ilizarov method does not ensure the complete normalization of ankle joint kinematic parameters. Therefore, intense personalized rehabilitation of the ankle joint is recommended.

## Figures and Tables

**Table 1:** Differences in hip flexion and hip abduction 24–48 month after Ilizarov therapy.

	Patients after Surgery (*n* = 23)
Minimum hip flexion, OL [°]	−12.4 [(−23.80)–(−4.0)]
Minimum hip flexion, NOL [°]	−13.3 [(−19.70)–(−2.8)]
*p*-value	0.911
Maximum hip flexion, OL [°]	29.7 (21.3–40.9)
Maximum hip flexion, NOL [°]	31.6 (10.8–46.7)
*p*-value	0.551
Hip flexion range, OL [°]	42.0 (29.0–57.9)
Hip flexion range, NOL [°]	44.3 (20.9–60.5)
*p*-value	0.632
Minimum hip abduction, OL [°]	−8.1 [(−14.1)–(−0.7)]
Minimum hip abduction, NOL [°]	−8.3 [(−17.4)–(−2.7)]
*p*-value	0.746
Maximum hip abduction, OL [°]	7.1 (1.8–17.4)
Maximum hip abduction, NOL [°]	7.2 (0.1–17.5)
*p*-value	0.575
Hip abduction range, OL [°]	15.3 (10.3–27.3)
Hip abduction range, NOL [°]	16.9 (8.1–24.6)
*p*-value	0.328

Data are medians and 5th–95th percentiles; NOL, non-operated limb; OL, operated limb.

**Table 2:** Differences in knee flexion after Ilizarov therapy.

	Patients after Surgery (*n* = 23)
Minimum knee flexion, OL [°]	−3.0 [(−7.5)–(−0.1)]
Minimum knee flexion, NOL [°]	−0.5 [(−20.4)–0.1]
*p*-value	0.127
Maximum knee flexion, OL [°]	58.6 (38.0–72.9)
Maximum knee flexion, NOL [°]	63.5 (21.6–74.7)
*p*-value	0.878
Knee flexion range, OL [°]	63.5 (44.2–74.9)
Knee flexion range, NOL [°]	64.9 (32.8–75.7)
*p*-value	0.809

Data are medians and 5th–95th percentiles; NOL, non-operated limb; OL, operated limb.

**Table 3:** Differences in ankle dorsiflexion, inversion, and abduction after Ilizarov therapy.

	Patients after Surgery (*n* = 23)
Minimum ankle dorsiflexion, OL [°]	−14.3 [(−33.5)–(−3.5)]
Minimum ankle dorsiflexion, NOL [°]	−23.2 [(−32.8)–(−8.4)]
*p*-value	**0.011**
Maximum ankle dorsiflexion, OL [°]	7.2 (2.2–16.8)
Maximum ankle dorsiflexion, NOL [°]	12.8 (4.5–24.4)
*p*-value	**<0.001**
Ankle dorsiflexion range, OL [°]	21.3 (8.6–41.8)
Ankle dorsiflexion range, NOL [°]	34.3 (22.4–47.1)
*p*-value	**<0.001**
Minimum ankle inversion, OL [°]	−4.8 [(−16.2)–(−1.0)]
Minimum ankle inversion, NOL [°]	−7.4 [18.8–(−4.5)]
*p*-value	**0.005**
Maximum ankle inversion, OL [°]	7.2 (0.7–15.8)
Maximum ankle inversion, NOL [°]	11.9 (1.3–33.7)
*p*-value	**0.011**
Ankle inversion range, OL [°]	11.3 (4.3–26.9)
Ankle inversion range, NOL [°]	20.7 (7.6–42.1)
*p*-value	**<0.001**
Minimum ankle abduction, OL [°]	−8.0 [(−28.40–(−2.4)]
Minimum ankle abduction, NOL [°]	−13.2 [(−33.1)–(−6.0)]
*p*-value	**0.004**
Maximum ankle abduction, OL [°]	4.4 (0.8–17.2)
Maximum ankle abduction, NOL [°]	6.8 (2.7–13.0)
*p*-value	0.051
Ankle abduction range, OL [°]	13.9 (4.4–32.9)
Ankle abduction range, NOL [°]	19.7 (9.6–37.2)
*p*-value	**0.003**

Data are medians and 5th–95th percentiles; NOL, non-operated limb; OL, operated limb; Bold typeface indicates statistically significant differences.

## Data Availability

The datasets used and/or analyzed during the current study are available from the corresponding author on reasonable request. The data are not publicly available due to privacy.
